# Supplementary material for: Clinical Utility and Limitation of Diagnostic Ability for Different Degrees of Dysplasia of Intraductal Papillary Mucinous Neoplasms of the Pancreas Using 18F-Fluorodeoxyglucose-Positron Emission Tomography/Computed Tomography
Source: Cancers (Basel). 2021 Sep 15;13(18):4633. doi: 10.3390/cancers13184633 (PMC8465733; doi:10.3390/cancers13184633)
Supplement: Supplementary file 1 [file cancers-13-04633-s001.zip › cancers-1346147-supplementary figure.pdf]

Supplementary Material: Clinical Utility and Limitation of Diagnostic Ability for Different Degrees of Dysplasia of Intraductal Papillary Mucinous Neoplasms of the Pancreas Using ^{18}F-Fluorodeoxyglucose-Positron Emission Tomography/Computed Tomography

Yuto Hozaka, Hiroshi Kurahara, Hideyuki Oi, Tetsuya Idichi, Yoichi Yamasaki, Yota Kawasaki, Kiyonori Tanoue, Megumi Jinguji, Masatoyo Nakajo, Atsushi Tani, Akihiro Nakajo, Yuko Mataki, Yoshihiko Fukukura, Hirotsugu Noguchi, Michiyo Higashi, Takashi Yoshiura, Akihide Tanimoto and Takao Ohtsuka

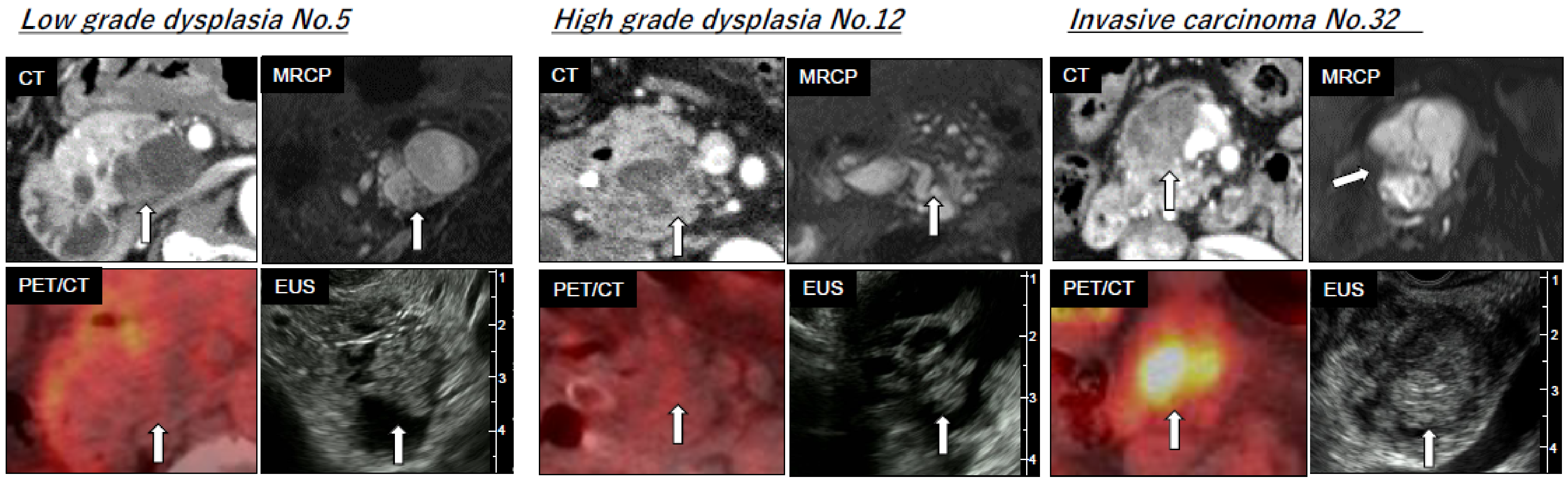

Figure S1. Representative MRI, EUS, and PET/CT images for IPMNs with LGD, HGD, and INV.
